# The Delivery of Person-Centered Care for People Living With Dementia in Residential Aged Care: A Systematic Review and Meta-Analysis

**DOI:** 10.1093/geront/gnad052

**Published:** 2023-05-05

**Authors:** Danielle Berkovic, Ann Macrae, Hannah Gulline, Phillipa Horsman, Sze-Ee Soh, Helen Skouteris, Darshini Ayton

**Affiliations:** Health and Social Care Unit, School of Public Health and Preventive Medicine, Monash University, Melbourne, Victoria, Australia; Mission & Corporate Development, Baptcare, Melbourne, Victoria, Australia; Health and Social Care Unit, School of Public Health and Preventive Medicine, Monash University, Melbourne, Victoria, Australia; Service Strategy Manager, Baptcare, Melbourne, Victoria, Australia; Health and Social Care Unit, School of Public Health and Preventive Medicine, Monash University, Melbourne, Victoria, Australia; Department of Physiotherapy, School of Primary and Allied Health Care, Monash University, Melbourne, Victoria, Australia; Health and Social Care Unit, School of Public Health and Preventive Medicine, Monash University, Melbourne, Victoria, Australia; Monash Warwick Professor in Health and Social Care Improvement and Implementation Science, Melbourne, Victoria, Australia; Health and Social Care Unit, School of Public Health and Preventive Medicine, Monash University, Melbourne, Victoria, Australia

**Keywords:** Cognitive decline, Intervention studies, Mixed methods, Older adults

## Abstract

**Background and Objectives:**

Person-centered care is the gold standard of care for people living with dementia, yet few systematic reviews have detailed how it is delivered in practice. This mixed-methods review aimed to examine the delivery of person-centered care, and its effectiveness, for people living with dementia in residential aged care.

**Research Design and Methods:**

A systematic review and meta-analysis. Eligible studies were identified across 4 databases. Quantitative and qualitative studies containing data on person-centered care delivered to people with dementia living in residential aged care were included. Meta-analysis using a random-effects model was conducted where more than 3 studies measured the same outcome. A narrative meta-synthesis approach was undertaken to categorize verbatim participant quotes into representative themes. Risk of bias was undertaken using quality appraisal tools from the Joanna Briggs Institute.

**Results:**

41 studies were identified for inclusion. There were 34 person-centered care initiatives delivered, targeting 14 person-centered care outcomes. 3 outcomes could be pooled. Meta-analyses demonstrated no reduction in agitation (standardized mean difference −0.27, 95% confidence interval [CI], −0.58, 0.03), improvement in quality of life (standardized mean difference −0.63, 95% CI: −1.95, 0.70), or reduced neuropsychiatric symptoms (mean difference −1.06, 95% CI: −2.16, 0.05). Narrative meta-synthesis revealed barriers (e.g., time constraints) and enablers (e.g., staff collaboration) to providing person-centered care from a staff perspective.

**Discussion and Implications:**

The effectiveness of person-centered care initiatives delivered to people with dementia in residential aged care is conflicting. Further high-quality research over an extended time is required to identify how person-centered care can be best implemented to improve resident outcomes.

The number of people with dementia is expected to increase from 57.4 million cases worldwide in 2019 to 152.8 million cases by 2050 (GBD 2019 Dementia Forecasting Collaborators, 2022). Dementia is prevalent among those living in residential aged care (RAC): 48% in the United States (Centers for Disease Control and Prevention, 2022), 54% in Australia (Australian Institute of Health and Welfare, 2021), and 70% in the United Kingdom (Alzheimer’s Research UK, 2013).

People living with dementia in RAC may have specific care needs that differ from those in RAC who do not have dementia, particularly around activities of daily living, cognition issues, and managing behavioral and psychological symptoms (Moyle et al., 2015). The Australian Royal Commission into Aged Care, which was established in 2018 to inquire into the quality and safety of aged care, found “many examples of inexpert dementia care caused unnecessary distress” (Royal Commission into Aged Care Quality and Safety, 2021). The World Health Organization’s action plan on the public health response to dementia suggests the implementation of person-centered care in RAC to improve current gaps in care (World Health Organization, 2017). This was also a key recommendation from the Australian Royal Commission into Aged Care (Royal Commission into Aged Care Quality and Safety, 2021).

Person-centered care is considered the gold standard of care for people living with dementia (American Geriatrics Society Expert Panel on Person-Centered Care, 2016). Key dimensions of person-centered care include physical, mental, and emotional support, tailored communication, involvement of carers and family, and access to care (Australian Commission on Safety and Quality in Health Care, 2022). Within RAC, person-centered care can reduce agitation (Ballard et al., 2018), neuropsychiatric symptoms (Rokstad et al., 2013), depression (Markle-Reid et al., 2011), and improve quality of life (Chenoweth et al., 2009) for people living with dementia. Person-centered care delivery is also associated with improved staff perception of job strain (Barbosa et al., 2015). Despite the demonstrated benefits of person-centered care, no reviews have thoroughly examined its application in RAC for people living with dementia.

A 2022 systematic review and meta-analysis on this topic were restricted to interventional studies (Lee et al., 2022). A 2019 systematic review restricted their inclusion criteria to four person-centered care outcomes (function, mood, neuropsychiatric symptoms, and quality of life; Chenoweth et al., 2019), omitting the opportunity to examine other important outcomes in the context of dementia care. A 2017 systematic review found that person-centered care interventions promote positive outcomes for people living with dementia, but these findings were not specific to RAC (Kim & Park, 2017). These reviews synthesize quantitative data on the effectiveness of person-centered care in RAC, but it is important to examine qualitative data which may explain findings to date. This review will provide the most up-to-date quantitative and qualitative evidence on the delivery of person-centered care, and its effectiveness, for people with dementia in RAC.

Specific objectives were to:

1. Determine the different frameworks, models of care, and programs currently used in the delivery of person-centered care;
2. Identify what type of person-centered care, and outcomes achieved, were delivered within the identified frameworks, models of care, and programs;
3. Examine the effect of delivering person-centered care to people with dementia living in RAC on outcomes identified in Objective 2; and
4. Explore the barriers and enablers to providing and receiving person-centered care, from the perspective of RAC residents and staff.

## Method

### Design

A systematic review and meta-analysis were undertaken (PROSPERO International Prospective Register of Systematic Reviews registration number 106919). The review is reported according to the Preferred Reporting Items for Systematic Reviews and Meta-Analysis (PRISMA) statement (Supplementary File 1).

### Deviations From Protocol

Because registering this review on PROSPERO, some changes have been made to the research objectives and methods. The three research questions listed on PROSPERO have been replaced with the four objectives listed earlier. We removed Organization for Economic Co-operation and Development (OECD) countries from the inclusion criteria. Review Manager V.5.0 was used for data synthesis, and not STATA V16.

### Search Strategy

An electronic literature search was undertaken in Medline, PsycINFO, Embase, and CINAHL databases (up to December 2021, Supplementary File 2). The reference lists of key literature and systematic reviews identified in the initial search yield were reviewed for additional primary studies. The search strategy was limited to the English language with no timeframe limit placed on published studies. The search strategy did not include gray literature, nonprimary data, or systematic reviews to ensure that the review was manageable and that studies addressed the research objectives.

### Study Selection

Eligible studies were primary quantitative, qualitative, or mixed-methods design studies that reported on person-centered care for people living with dementia in RAC. All dementia subtypes were eligible for inclusion. Diagnoses such as cognitive impairment were also eligible for inclusion, as long as it was specified that this impairment stemmed from a dementia diagnosis. Studies were excluded if they did not address person-centered care, outcomes reported did not occur in RAC, diagnostic criteria were not clear, or the full text was not available in English or unavailable in its entirety.

Two reviewers (D. Berkovic, A. Macrae) independently screened titles and abstracts of retrieved studies using Covidence (Veritas Health Innovation Ltd, Melbourne, Australia)—a web-based software that assists researchers to screen references and undertake data extraction for systematic reviews and meta-analyses—to determine eligibility. All potentially eligible studies were reviewed independently at the full-text stage (D. Berkovic, A. Macrae). At each review stage, discordance regarding eligibility was discussed and resolved through consensus.

### Data Extraction

Three reviewers (D. Berkovic, A. Macrae, H. Gulline) independently extracted data using a customized template. Data extracted included the study design, country, diagnosis, gender, age, and relevant outcomes concerning the delivery of person-centered care to people living with dementia in RAC.

### Outcome Measures and Qualitative Themes

As there are multiple definitions of person-centered care, all person-centered care definitions, models of care, and frameworks were included. Qualitative results emerged through second-order author-derived themes and were categorized through first-order examination of direct quotes.

### Risk of Bias Assessment

Two reviewers (D. Berkovic, H. Gulline) assessed the quality of included studies using validated critical appraisal tools from the Joanna Briggs Institute (JBI; The University of Adelaide, 2022). The Mixed Methods Appraisal Tool (MMAT) was used to assess mixed-methods studies (Hong et al., 2021).

The JBI critical appraisal tools included 8–13 items depending on the study design. Scores were converted to percentages to allow for the comparison of evidence quality scores across different study types (the higher the score of the study, the less bias present). The JBI advises that studies should not be included in the analysis if they are of low quality (score ≤50%). We included all moderate (51%–79%) and good-quality studies (80%–100%). Moderate studies are likely to have moderate but justifiable levels of bias, and good-quality studies are likely to contain little bias.

The MMAT is a critical appraisal tool designed for use across mixed-methods studies. The MMAT asks five questions relating to the rationale for using mixed methods to address the research aims. Response options to each question include “yes,” “no,” or “can’t tell.” If a study achieved a “yes” for four to five questions, it was rated as high quality; if a study achieved a “yes” for three questions, it was rated as medium quality, and a study that achieved less than three yes, it was classified as low quality. The MMAT user guide does not state that studies rated as low quality should be excluded. As such, all studies assessed using the MMAT were included (Supplementary File 3).

Two reviewers (D. Berkovic, H. Gulline) independently conducted the quality assessment. For scores with ≤10% difference, the average score between the reviewers was used. Where there was stronger disagreement, the study was assessed in tandem, and a consensus score was derived. Where agreement counts not be reached, a third reviewer (D. Ayton) was consulted.

### Data Synthesis and Meta-Analysis of Quantitative Studies

Study characteristics and demographic data were reported using mean (standard deviation [*SD*]), median (interquartile range [IQR]), or frequency. Meta-analysis was conducted where more than three studies measured the same outcome (Borenstein et al., 2021). Experimental and quasi-experimental studies that contained a control group, and aimed to determine the effect of person-centered care interventions on all potential resident outcomes, were eligible for meta-analysis inclusion (Nestoriuc et al., 2008). Sensitivity analysis was conducted where studies were removed based on their design to ensure that those with a high risk of bias did not affect meta-analysis results.

The effect estimate reported for each intervention group was included separately within the meta-analysis to include studies with more than one intervention arm but only one control group. In this instance, the number of participants in the control group was divided equally between the comparisons as per the method described by Cochrane (Higgins et al., 2022).

All outcomes included in the meta-analysis reported continuous data but used varying outcome measures. For example, agitation was measured using the Cohen–Mansfield Agitation Inventory and the Behavioral Activity Rating Scale, and quality of life was measured using the Quality of Life in Late Stage Dementia (QUALID) Scale, and the Alzheimer’s Disease-Related Quality of Life (ADRQL) Instrument. Hence, we used the standardized mean difference as our summary statistic.

Mean difference and 95% confidence interval (CI) were used to describe the treatment effect. Statistical heterogeneity was assessed using the *I*^2^ statistic, and a high degree of statistical heterogeneity was present if *I*^2^ values were greater than 50%. A random-effects model was applied in this instance. It was anticipated that a random-effects model would be required across all meta-analyses due to the diverse RAC residents. All analyses were conducted with the use of Review Manager V.5.0 (Revman, The Cochrane Collaboration; Oxford, UK).

### Data Synthesis of Qualitative Studies

A narrative meta-synthesis approach was undertaken to categorize verbatim participant quotes into themes to facilitate an examination of outcomes based on primary data (Edwards & Kaimal, 2016).

## Results

### Study Selection and Inclusion

There were 1,099 articles identified (67 duplicates). After reviewing titles and abstracts, 898 articles were excluded, leaving 133 articles for full-text review. The full-text review process yielded 51 articles for quality and risk of bias assessment. Ten articles were deemed to be of low methodological quality and were excluded from the review. The full study selection and inclusion process are shown in Supplementary File 1.

### Study Characteristics

Forty-one studies from 13 different countries: 7 from Australia, 2 from Belgium, 5 from Canada, 1 from Denmark, 3 from Germany, 1 from Japan, 2 from the Netherlands, 3 from Norway, 4 from Portugal, 1 from Sweden, 3 from the United Kingdom, 8 from the United States; and 1 study conducted across Belgium, Norway, Portugal, and Romania with a wide range of person-centered care-related interventions and resident outcomes were included. The included studies were published from 1996 to 2021. Of the 41 studies, 15 were randomized-controlled trials (RCTs), 10 adopted a quasi-experimental methodology, 8 adopted a mixed-methods design, and 8 adopted a qualitative methodology.

All but one study reported on the number of included RAC sites, ranging from 1 (where a qualitative case study was conducted) to 69. All studies also reported the number of residents or staff, depending on the target population group. The smallest sample was 4 staff members (where the same qualitative case study was conducted), but one mixed-methods study included 452 staff members. The smallest number of residents included in the studies, where residents were the target population, was 16; the highest number of residents was 847.

Across the majority of studies, participants were primarily female (64%–100%). Three studies did not report on participants’ sex, and two studies reported majority male participants. Almost all studies included participants with a dementia diagnosis; five included participants with Alzheimer’s Disease, one study included participants with cognitive impairment, and one study included participants with either moderate to severe dementia, Alzheimer’s, vascular dementia, mixed vascular dementia and Alzheimer’s, and Lewy body dementia. Study characteristics for all included studies can be found in Table 1.

**Table 1.** Characteristics of Included Studies

Author(s), year	Study design	Person-centered framework, model, or program	Number RAC services	Number of residents	Number of staff	Country	% Female	Participant diagnosis
**Ballard et al. (2016)**	Cluster factorial RCT	Well-being and Health for People with Dementia (WHELD)	69	277	138	United Kingdom	71	Dementia
**Ballard et al. (2018)**	Cluster RCT	WHELD	16	847	NS	United Kingdom	74	Dementia
**Barbosa et al. (2016)**	Pretest post-test	Researcher-designed person-centered care intervention based on psychoeducation and multisensory stimulation	4	NS	56	Portugal	100	Dementia
**Barbosa et al. (2016)**	Pretest post-test	Researcher-designed person-centered care intervention based on psychoeducation and multisensory stimulation	4	47	53	Portugal	100	Dementia
**Barbosa et al. (2017a)**	Pretest post-test	Researcher-designed person-centered care intervention based on psychoeducation and multisensory stimulation	4	0	23	Portugal	100	Dementia
**Barbosa et al. (2017b)**	Process evaluation	Researcher-designed person-centered care intervention based on psychoeducation and multisensory stimulation	4	45	56	Portugal	100	Dementia
**Berendonk et al. (2019)**	Cluster RCT	Mini-interventions for meaningful situations for people with dementia (DEMIAN)	20	84	180	Germany	Experimental group: 87.5 Control group: 85.3	Dementia
**Boersma et al. (2017)**	Mixed-methods pretest post-test, interviews, focus groups	Veder Contact Method	4	0	228	The Netherlands	96	Dementia
**Booth et al. (2020)**	Qualitative case study	Positive Interaction Engagement (PIE) Program	1	0	4	Australia	85.7	Dementia
**Burack et al. (2012)**	Interventional	Researcher-designed culture change	13	101	0	United States	64	Cognitive impairment
**Chenoweth et al. (2009)**	Cluster RCT	Researcher-designed person-centered care program and Dementia Care Mapping	15	289	20	Australia	NS	Dementia
**Chenoweth et al. (2015)**	Mixed-methods (surveys, interviews, evaluation care plans)	Person-Centered Dementia Care and Environment (PerCEN)	NS	73 (proxy family members)	99	Australia	DCM: 83 PCC: 76 UC: 73	Dementia
**Chu et al. (2020)**	Quasi-experimental time series	Multifaceted Walking Intervention (MWI)	2	26	0	Canada	80.8	Dementia
**Ducak et al. (2018)**	Qualitative (interviews)	Montessori	1	0	17	Canada	100	Dementia
**Froggatt et al. (2020)**	Feasibility cluster RCT	Namaste Care	8	32	97	England	47	Dementia
**Gillis et al. (2019)**	Pretest post-test	ABC Method: Antecedent Events, Target Behaviors, Consequent Events, and the Senses Framework	3	71	0	Belgium	68	Dementia
**Goodall (2021**)	Qualitative (participant observation and interviews)	SENSE-GARDEN	4	0	8	Norway, Belgium, Portugal, Romania	100	Dementia
**Goossens (2020**)	Pretest post-test RCT	“We Decide” – We Discuss End-of-Life Choices	65	0	311	Belgium	87.5	Dementia
**Halek et al. (2020)**	Stepped wedge cluster RCT	WELCOME-Innovative dementia-oriented assessment system (WELCOME-Ida) and Welcome-Narrative Approach (WELCOME-NEO)	6	224	189	Germany	Welcome-IdA: 65 Welcome-NEO: 73	Dementia
**Hebert et al. (2018)**	Mixed-methods (interventional, interviews, field notes)	Individualized music	1	22	6	United States	53	Dementia
**Hoeffer et al. (2006)**	RCT	Researcher-designed, person-centered showering and bathing techniques	15	69	37	United States	94.6	Alzheimer’s or related dementia
**Jacobsen et al. (2017)**	Mixed-methods (cluster RCT, participatory action research, ethnography)	Researcher-designed MEDCED intervention around using person-centered measures to avoid use of restraints	24	0	452	Norway	NS	Dementia
**Jeon et al. (2012)**	Cluster RCT	Researcher-designed person-centered care program and Dementia Care Mapping	15	0	194	Australia	85.5	Dementia
**Kontos et al. (2010)**	Qualitative (interviews, focus groups)	Modified Mitchell and Bournes Curriculum (1998), involving a drama-based educational intervention to introduce the concept of selfhood to dementia practitioners	2	0	24	Canada	91.7	Dementia
**Kontos et al. (2016)**	Before and after study	Elder clowning	2	23	0	Canada	69.6	- Moderate to severe dementia - Alzheimer’s - Vascular dementia -Mixed vascular dementia - Lewy Body
**Matthews et al. (1996)**	Longitudinal time design	Researcher-designed client-oriented care approach which prioritized autonomous decision making	1	33	0	Australia	63.6	Dementia
**Passalacqua and Harwood (2012)**	Pretest post-test	Dawn Brooker’s four elements of person-centered dementia care: valuing people, individualized care, personal perspectives, and the social environment	1	0	26	United States	89	Alzheimer’s
**Quasdorf et al. (2017)**	Convergent parallel mixed-methods (intervention and interviews)	Dementia Care Mapping	9	217	112	Germany	NS	Dementia
**Resnick et al. (2021)**	RCT	Function Focused Care for Assisted Living Using the Evidence Integration Triangle (FFC-AL-EIT)	59	550	0	United States	69	Dementia
**Roberts et al. (2015)**	Mixed methods (interviews and surveys)	ABLE Model of Care: Abilities and capabilities of the resident, Background of the resident, Leadership, education training and organizational culture change, physical Environment	1	16	18	Australia	75	Dementia
**Rokstad et al. (2013)**	Three-armed cluster RCT	Dementia Care Mapping, VIPS Practice Model	14	624	0	Norway	71.8	Dementia
**Rosvik (2014**)	RCT	VIPS Practice Model	14	624	0	Norway	71	Dementia
**Sloane et al. (2004)**	RCT	Nonpharmacological techniques of person-centered showering and bathing	15	69	37	United States	85.8	Alzheimer’s
**Swall et al. (2020)**	Qualitative	Caregiver singing or music therapy caregiving	3	0	30	Sweden	96.7	Dementia
**Thoft et al. (2021)**	Qualitative	Life story work using the digital My Life Story app	1	0	8	Denmark	87.5	Dementia
**van der Ploeg et al. (2013)**	RCT	Montessori	9	57	0	Australia	68.2	Dementia
**van Haitsma et al. (2015)**	RCT	Researcher-selected individualized positive psychosocial interventions, including physical exercise, singing, reminiscence, ADLs, sensory stimulation, etc.	1	180	0	United States	82.2	Dementia
**van Weert et al. (2006)**	Pretest post-test	Snoezelen	6	61	80	Netherlands	80.6	Dementia
**Williams et al. (2013)**	Mixed methods (intervention, interviews, focus groups)	P.I.E.C.E.S. Program: Physical Intellectual, Emotional, Capabilities, Environmental, and Social	6	1194	43	Canada	Residents: 68 Staff: 93	Dementia
**Williams et al. (2018)**	Secondary analysis of video recordings	Changing Talk (CHAT) communication training—elderspeak	11	39	49	United States	67	Alzheimer’s
**Yasuda et al, 2017**	Pretest post-test	Dementia Care Mapping	1	40	NS	Japan	77.5	Alzheimer’s

*Notes*: ADLs = activities of daily living; DCM = dementia care mapping; NS = not stated; PCC = person-centred care; RAC = residential aged care; RCT = randomized-controlled trial; UC = usual care; VIPS = Values people, Individual’s needs, Perspective of service user-understands care from the perspective of the person with dementia.

### Risk of Bias Assessment Results

Seventeen studies were rated as high quality (Ballard et al., 2016, 2018; Barbosa et al., 2017b; Barbosa, Nolan, et al., 2016; Boersma et al., 2017; Chenoweth et al., 2009; Ducak et al., 2018; Goodall et al., 2021; Goossens et al., 2020; Hebert et al., 2018; Jacobsen et al., 2017; Kontos et al., 2010; Quasdorf et al., 2017; Swall et al., 2020; Van Haitsma et al., 2015; van Weert et al., 2006; Williams et al., 2013), and 23 studies were rated as medium quality (Barbosa et al., 2017a; Barbosa, Marques, et al., 2016; Berendonk et al., 2019; Booth et al., 2020; Burack et al., 2012; Chenoweth et al., 2015; Chu et al., 2020; Froggatt et al., 2020; Gillis et al., 2019; Halek et al., 2013; Hoeffer et al., 2006; Jeon et al., 2012; Kontos et al., 2016; Matthews et al., 1996; Passalacqua & Harwood, 2012; Resnick et al., 2021; Rokstad et al., 2013; Rosvik et al., 2014; Sloane et al., 2004; Thoft et al., 2021; van der Ploeg et al., 2013; Williams et al., 2018; Yasuda & Sakakibara, 2017). Ten studies were rated as low quality and subsequently excluded. Roberts et al. (2015) was graded as low quality, but as stated in the MMAT user guide, low-quality mixed-methods studies should be not excluded from a review. As a result, this study was still included in this review. JBI quality and risk of bias results can be found in Supplementary File 4.

#### What are the different frameworks, models of care, and programs currently used in the delivery of person-centered care in RAC?

The included studies contained 34 different frameworks, models of care, and programs to deliver person-centered care to people in RAC. The frameworks were broadly categorized into two subtypes: validated methods of delivering person-centered care that have been trialed across multiple studies and by multiple researchers, and fit-for-purpose methods specific to one study.

There were 23 validated frameworks, models of care, or programs used to deliver person-centered care across the included studies (Ballard et al., 2016, 2018; Berendonk et al., 2019; Boersma et al., 2017; Booth et al., 2020; Chenoweth et al., 2009, 2015; Ducak et al., 2018; Froggatt et al., 2020; Gillis et al., 2019; Goodall et al., 2021; Goossens et al., 2020; Halek et al., 2013; Jeon et al., 2012; Kontos et al., 2010, 2016; Passalacqua & Harwood, 2012; Quasdorf et al., 2017; Resnick et al., 2021; Roberts et al., 2015; Thoft et al., 2021; van der Ploeg et al., 2013; van Weert et al., 2006; Williams et al., 2013, 2018; Yasuda & Sakakibara, 2017). **Dementia Care Mapping** was the most frequently adopted method, found across five studies (Chenoweth et al., 2009; Jeon et al., 2012; Quasdorf et al., 2017; Rokstad et al., 2013; Yasuda & Sakakibara, 2017). The **Montessori for Dementia and Ageing** model of care was the next most frequently adopted model, employed across four studies (Booth et al., 2020; Ducak et al., 2018; Roberts et al., 2015; van der Ploeg et al., 2013). Booth et al. used the **Positive Interaction Engagement Program** based on Montessori principles (Booth et al., 2020). Roberts et al. used the **ABLE Model of Care** based on Montessori’s principle of acknowledging people with dementia as autonomous adults (Roberts et al., 2015). The **VIPS Practice Model** was used across two studies (Rokstad et al., 2013; Rosvik et al., 2014), as was **Well-being and Health for People with Dementia** (WHELD; Ballard et al., 2016, 2018).

There were 11 fit-for-purpose, researcher-designed methods of delivering person-centered care found in this review (Barbosa et al., 2017a, 2017b; Barbosa, Marques, et al., 2016; Barbosa, Nolan, et al., 2016; Burack et al., 2012; Chenoweth et al., 2009; Chu et al., 2020; Hebert et al., 2018; Hoeffer et al., 2006; Jacobsen et al., 2017; Matthews et al., 1996; Sloane et al., 2004; Swall et al., 2020; Van Haitsma et al., 2015). Three of these methods focused on communication between staff and residents (Barbosa, Marques, et al., 2016; Barbosa, Nolan, et al., 2016; Chenoweth et al., 2009) and music therapy (Hebert et al., 2018; Swall et al., 2020; Van Haitsma et al., 2015), two focused on activities of daily living (Chu et al., 2020; Van Haitsma et al., 2015) and improving quality of life (Chu et al., 2020; Hebert et al., 2018). The four Barbosa et al. studies used the same intervention which focused on psychoeducation and multisensory stimulation (Barbosa et al., 2017a, 2017b; Barbosa, Marques, et al., 2016; Barbosa, Nolan, et al., 2016). Table 2 describes these studies in more detail.

**Table 2.** Person-Centered Care Frameworks, Models of Care, and Programs; Person-Centered Care Outcomes.

Author(s), year	Person-centered care framework, model, or program	Person-centered care components													
		Agitation	Antipsychotic medication use	Mobility	Activities of daily living	Quality of life	Staff burnout	Understanding dementia	Understanding person-centered care	Neuropsych symptoms	Shared decision making and communication	Person-centered care environment	Stimulation (multisensory, motor)	Staff training and caregiving	Staff job satisfaction
**Ballard et al. (2016)**	Well-being and Health for People with Dementia (WHELD)		✓					✓	✓	✓					
**Ballard et al. (2018)**	WHELD	✓				✓				✓					
**Barbosa et al. (2016)**	Researcher-designed person-centered care intervention based on psychoeducation and multisensory stimulation							✓	✓		✓	✓	✓	✓	
**Barbosa et al. (2016)**	Researcher-designed person-centered care intervention based on psychoeducation and multisensory stimulation							✓	✓		✓	✓	✓	✓	
**Barbosa et al. (2017a)**	Researcher-designed person-centered care intervention based on psychoeducation and multisensory stimulation							✓	✓		✓	✓	✓	✓	
**Barbosa et al. (2017a)**	Researcher-designed person-centered care intervention based on psychoeducation and multisensory stimulation							✓	✓		✓	✓	✓	✓	
**Berendonk et al. (2019)**	Mini interventions for meaningful situations for people with dementia (DEMIAN)							✓							✓
**Boersma et al. (2017)**	Veder Contact Method														✓
**Booth et al. (2020)**	Positive Interaction Engagement (PIE) Program											✓	✓		
**Burack et al. (2012)**	Researcher-designed culture change	✓													
**Chenoweth et al. (2009)**	Researcher-designed person-centered care program and Dementia Care Mapping	✓	✓			✓				✓					
**Chenoweth et al. (2015)**	Person-Centered Dementia Care and Environment (PerCEN)	✓										✓			
**Chu et al. (2020)**	Multifaceted Walking Intervention (MWI)			✓	✓	✓									
**Ducak et al. (2018)**	Montessori				✓							✓			
**Froggatt et al. (2020)**	Namaste Care	✓				✓				✓					
**Gillis et al. (2019**	ABC Method: Antecedent Events, Target Behaviors, Consequent Events, and the Senses Framework	✓								✓					
**Goodall (2021)**	SENSE-GARDEN											✓			
**Goossens (2020)**	“We Decide”—We Discuss End-of-Life Choices										✓				
**Halek et al. (2020)**	WELCOME-Innovative dementia oriented assessment system (WELCOME-Ida) and Welcome-Narrative Approach (WELCOME-NEO)					✓	✓			✓					
**Hebert et al. (2018)**	Individualized music							✓	✓					✓	
**Hoeffer et al. (2006)**	Researcher-designed, person-centered showering and bathing techniques								✓						
**Jacobsen et al. (2017)**	Researcher-designed MEDCED intervention around using person-centered measures to avoid use of restraints								✓		✓				
**Jeon et al. (2012)**	Researcher-designed person-centered care program and Dementia Care Mapping						✓			✓					
**Kontos et al. (2010)**	Modified Mitchell and Bournes Curriculum (1998), involving a drama-based educational intervention to introduce the concept of selfhood to dementia practitioners								✓		✓				
**Kontos et al. (2016)**	Elder clowning	✓				✓				✓				✓	
**Matthews et al. (1996)**	Researcher-designed client-oriented care approach which prioritized autonomous decision making	✓													
**Passalacqua and Harwood (2012)**	Dawn Brooker’s four elements of person-centered dementia care: valuing people, individualized care, personal perspectives, and the social environment						✓				✓				
**Quasdorf et al. (2017)**	Dementia Care Mapping					✓									
**Resnick et al. (2021)**	Function Focused Care for Assisted Living Using the Evidence Integration Triangle (FFC-AL-EIT)	✓									✓				
**Roberts et al. (2015)**	ABLE Model of Care: Abilities and capabilities of the resident, Background of the resident, Leadership, education training and organizational culture change, physical Environment	✓	✓			✓						✓			
**Rokstad et al. (2013)**	Dementia Care Mapping, VIPS Practice Model	✓				✓				✓					
**Rosvik (2014)**	VIPS Practice Model									✓					
**Sloane et al. (2004)**	Nonpharmacological techniques of person-centered showering and bathing	✓													
**Swall et al. (2020)**	Caregiver singing or music therapy caregiving								✓		✓				
**Thoft et al. (2021)**	Life story work using the digital My Life Story app								✓						
**van der Ploeg et al. (2013)**	Montessori	✓						✓							
**van Haitsma et al. (2015)**	Researcher-selected individualized positive psychosocial interventions, including physical exercise, singing, reminiscence, ADLs, sensory stimulation, etc.				✓										
**van Weert et al. (2006)**	Snoezelen												✓	✓	
**Williams et al. (2013)**	P.I.E.C.E.S. Program: Physical Intellectual, Emotional, Capabilities, Environmental, and Social				✓				✓						
**Williams et al. (2018)**	Changing Talk (CHAT) communication training—elderspeak										✓				
**Yasuda et al. (2017)**	Dementia Care Mapping					✓								✓	

*Notes:* ABLE = Abilities and capabilities of the resident, Background of the resident, Leadership, education training and organizational culture change, physical Environment; ADLs = activities of daily living; VIPS = Values people, Individual’s needs, Perspective of service user-understands care from the perspective of the person with dementia.

#### What types of person-centered care, and outcomes achieved, were delivered within the identified frameworks, models of care, and programs?

The included studies contained 14 various elements of person-centered care. Outcomes include agitation, antipsychotic medication use, mobility, activities of daily living, quality of life, understanding dementia, understanding person-centered care, neuropsychiatric symptoms, person-centered care and dementia-friendly environments, multisensory and motor stimulation, staff burnout, staff training and caregiving, and staff job satisfaction.

Eleven quantitative studies assessed agitation (Ballard et al., 2018; Burack et al., 2012; Chenoweth et al., 2009; Gillis et al., 2019; Kontos et al., 2016; Matthews et al., 1996; Resnick et al., 2021; Roberts et al., 2015; Rokstad et al., 2013; Sloane et al., 2004; van der Ploeg et al., 2013). Agitation was reported to improve among residents in almost all studies using validated models (results ranging from *p* = .05 to *p* < .001; Gillis et al., 2019; Sloane et al., 2004), yet in one cluster RCT, where residents were assigned to the dementia care mapping group, agitation did not improve (*p* = .77; Chenoweth et al., 2009). Agitation also did not improve across studies utilizing researcher-designed, fit-for-purpose interventions. For example, Burack et al., who implemented a culture change program, found no significant change in verbal agitation among residents (*p* = .061).

Ten quantitative studies assessed the quality of life (Ballard et al., 2018; Chenoweth et al., 2009; Chu et al., 2020; Froggatt et al., 2020; Halek et al., 2013, 2020; Hoeffer et al., 2006; Kontos et al., 2016; Rokstad et al., 2013; Yasuda & Sakakibara, 2017). Descriptively, quality of life was reported to improve among residents in all but one study that included pre-existing, validated modes of delivering person-centered care (results ranging from *p* = .04 to *p* < .001; Kontos et al., 2016; Rokstad et al., 2013). Only in Halek et al. were there no reported differences in quality of life between participants in the intervention and control groups (Halek et al., 2013).

Ten quantitative studies assessed neuropsychiatric symptoms (Ballard et al., 2016, 2018; Chenoweth et al., 2009; Froggatt et al., 2020; Gillis et al., 2019; Halek et al., 2020; Jeon et al., 2012; Kontos et al., 2016; Rokstad et al., 2013; Rosvik et al., 2014). Five studies reported improvements in neuropsychiatric symptoms among residents, particularly for those participating in the WHELD intervention, with symptoms improving significantly in both Ballard et al. studies (*p* = .05; Ballard et al., 2016, and *p* < .0001; Ballard et al., 2018). Elder clowning had a similarly significant effect on reducing residents’ neuropsychiatric symptoms (*p* = .01; Kontos et al., 2016). Both Rokstad et al. and Rosvik et al. using the VIPS Practice Model demonstrated a significant reduction in neuropsychiatric symptoms (*p* = .04; Rokstad et al., 2013, 2014). Two studies found no significant change in residents’ neuropsychiatric symptoms despite using validated models (Chenoweth et al., 2009; Gillis et al., 2019).

Eleven quantitative studies assessed shared decision making and communication (Barbosa et al., 2017a, 2017b; Barbosa, Marques, et al., 2016; Barbosa, Nolan, et al., 2016; Goossens et al., 2020; Jacobsen et al., 2017; Kontos et al., 2010; Passalacqua & Harwood, 2012; Resnick et al., 2021; Swall et al., 2020; Williams et al., 2018). In both Barbosa et al. studies, interventions led to increased staff engagement with residents, such as laughing with residents (*p* < .001; Barbosa, Marques, et al., 2016; Barbosa, Nolan, et al., 2016). Staff were also statistically more likely to participate in social conversations with residents as a result of the intervention. Staff being taught dementia-friendly communication strategies were more likely to submit reports detailing concerns about residents’ emotional well-being (*p* = .027; Berendonk et al., 2019). Passalacqua and Harwood also measured dementia-friendly communication components of asking yes/no questions (*p* < .05), giving choice (*p* < .05), and using humor (*p* < .01) with residents, all of which produced significant results (Passalacqua & Harwood, 2012). Williams et al. reduced the mean percentage of time that staff used elderspeak to communicate with residents from 28.5 to 19.6 (*p* = .002; Williams et al., 2018).

The various quantitative studies delivered person-centered care over different periods, ranging from 4 weeks to 2 years. Fourteen studies collected data only from people living with dementia in RAC, nine studies collected data only from staff working in RAC, six studies collected data from both people living with dementia in RAC and staff working in RAC, and two studies collected data via other methods, for example, family members. Supplementary File 5 describes these findings in more detail.

#### Meta-analysis

Three outcomes were included in the meta-analysis: agitation, neuropsychiatric symptoms, and quality of life. Very high *I*^2^ values were observed for all three outcomes indicating a high degree of statistical heterogeneity. A random-effects model was, therefore, adopted for all meta-analyses based on the assumption that the effects of person-centered care vary between studies. John Fletcher’s four questions to consider for heterogeneity (Fletcher, 2007) were discussed among the authorship team before progressing with the meta-analysis. Based on the pressing need to better deliver person-centered care to people living with dementia in RAC, and to try and understand what currently does and does not work in this setting, the authorship team agreed to proceed with the meta-analysis.

The delivery of person-centered care was not found to have a statistically significant impact on agitation, neuropsychiatric symptoms, or quality of life for people living with dementia in RAC. All studies included in agitation and neuropsychiatric symptoms meta-analyses were RCTs. As such, sensitivity analysis was not performed. One of the studies included in the quality-of-life meta-analysis was a nonrandomized quasi-experimental time series. We, therefore, performed a sensitivity analysis by removing this study to examine the impact of assumptions or unmeasured variables in the data.

##### Quality of Life.

**Figure 1.**
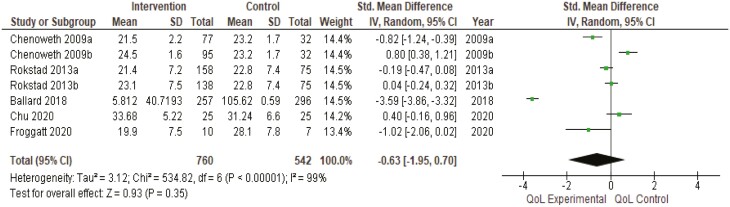
Quality of life meta-analysis results. CI = confidence interval; *I*^2^ = degree of heterogeneity; *SD* = standard deviation; Std. = standardized.

Data were only able to be pooled for five studies (1,375 residents, 108 RAC facilities). Two of these studies contained two intervention arms but only one control group. As described in the methods, due to multiple intervention arms, results are available for seven subgroups. Although four of these indicated that person-centered care interventions significantly improved the quality of life for people living with dementia in RAC, meta-analysis results, overall, were statistically insignificant for quality of life (standardized mean difference −0.63, 95% CI: −1.95, 0.70, *I*^2^ 99%). Results were still statistically insignificant after removing the quasi-experimental study from the meta-analysis (standardized mean difference −0.80, 95% CI: −2.26, 0.66, *I*^2^ 99%) (Figure 1).

##### Neuropsychiatric Symptoms.

**Figure 2.**
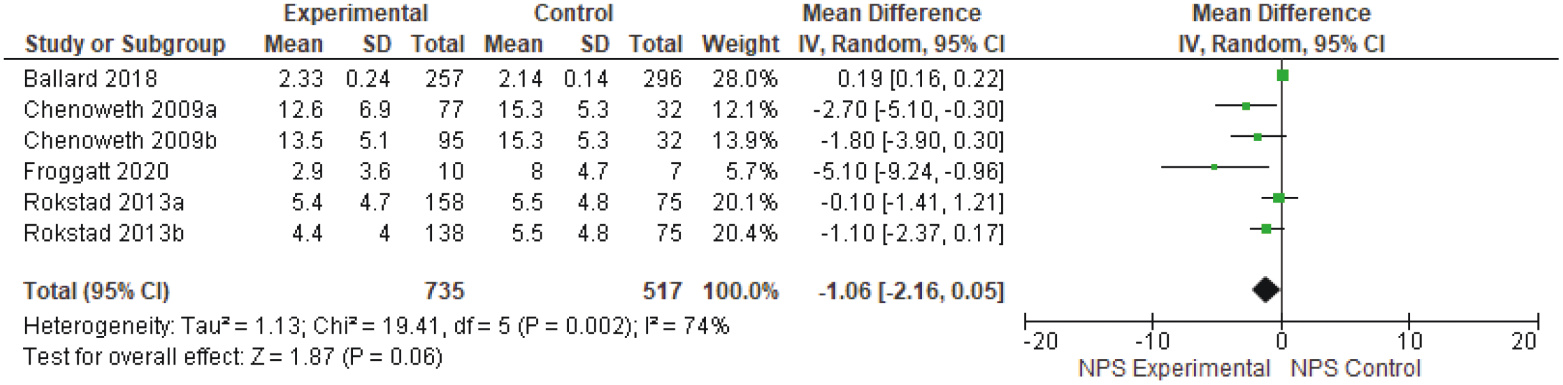
Neuropsychiatric symptoms meta-analysis results. CI = confidence interval; *I*^2^ = degree of heterogeneity; *SD* = standard deviation; Std. = standardized.

Data were only able to be pooled for four studies (1,349 residents, 106 RAC facilities). Similarly, due to multiple intervention arms across two studies, results are available for six groups. Half of the studies indicated that person-centered care interventions improved neuropsychiatric symptoms in people living with dementia in RAC, but these findings were not statistically significant overall (mean difference −1.06, 95% CI: −2.16, 0.05, *I*^2^ 74%; Figure 2).

##### Agitation.

**Figure 3.**
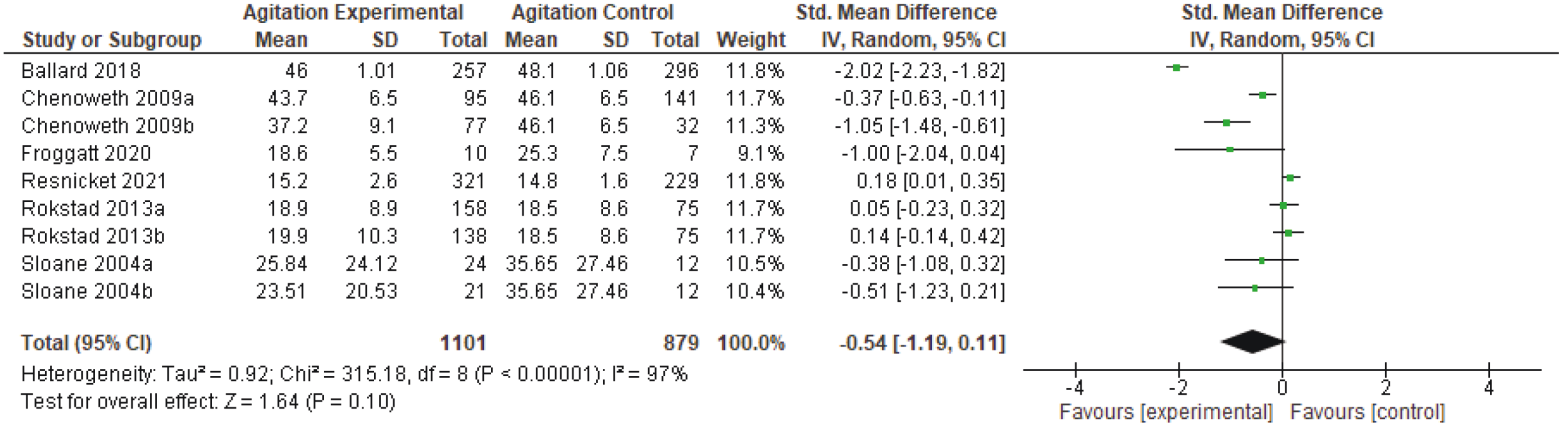
Agitation meta-analysis results. CI = confidence interval; *I*^2^ = degree of heterogeneity; *SD* = standard deviation; Std. = standardized.

Data were pooled for six studies that measured agitation (1,968 residents, 180 RAC facilities). Again, due to multiple intervention arms across three studies, results are available for nine groups. Five studies indicated that person-centered care interventions significantly improved agitation in people living with dementia in RAC, but these findings were not statistically significant overall (standardized mean difference −0.27, 95% CI: −0.58, 0.03, *I*^2^ 83%; Figure 3).

#### Qualitative meta-synthesis

##### Barriers to providing person-centered care.

Six qualitative or mixed-methods studies explored barriers to providing person-centered care. Additional supporting quotes are provided in Supplementary File 6.

Three studies explored time as a barrier to providing person-centered care (Boersma et al., 2017; Ducak et al., 2018; Kontos et al., 2010). Staff lamented that “sometimes you have to rush rush rush,” but recognized that rushing “affects the residents’ mood” (Kontos et al., 2010). Documentation of toileting and bathing schedules were brought up as examples of “how fast things have to get done” (Ducak et al., 2018). One study acknowledged the role that staff shortages play in trying to manage time: “our dementia unit is quite active right at the moment and at this point it’s attention span and staffing levels” (Ducak et al., 2018). The same study recognized that “the very task-oriented work environment” of RAC contributes to time constraints.

Two studies identified challenging resident behaviors as a barrier to providing person-centered care, citing “aggressive stuff on display” (Booth et al., 2020; Kontos et al., 2010). Staff revealed that they have had residents try to “spit on you, hit you, get aggressive” (Kontos et al., 2010), but others showed an understanding of why residents may display this type of anger: “if their needs are not met, they are frustrated, they feel bored, and they have no control” (Booth et al., 2020). One study described that “residents’ psychosocial health needs are ignored” (Goodall et al., 2021), and that “the psychological aspects are not taken very well care of… this causes a lot of problems” (Gillis et al., 2019).

Two studies had staff members describe family expectations as a barrier to delivering person-centered care (Ducak et al., 2018; Thoft et al., 2021). Staff lamented that they “receive a lot of resistance from certain families because they expect to have everyone singing and dancing all the time” but that this view is unrealistic: “do you do that in your own life?” (Ducak et al., 2018). Different staff reported similar frustrations but recognized that may be because family members are “worn out” (Thoft et al., 2021).

##### Enablers to providing person-centered care.—

Twelve qualitative or mixed-methods studies explored enablers to providing person-centered care. Further supporting quotes are provided in Supplementary File 6.

Getting to know the resident was highlighted across six studies as the biggest enabler, of person-centered care (Booth et al., 2020; Goodall et al., 2021; Kontos et al., 2010; Roberts et al., 2015; Swall et al., 2020; Thoft et al., 2021). Staff explained that “it’s important to know the residents’ life stories, otherwise you don’t know how to help them” (Thoft et al., 2021). This sentiment was summed up across other studies, with participants stating that “knowing the interests of the residents prior to them having dementia does help” (Kontos et al., 2010), and that it’s important to get to know residents so that their RAC facility “feels like home” (Roberts et al., 2015).

Staff collaboration was identified across seven studies as an enabler to providing person-centered care (Chenoweth et al., 2015; Ducak et al., 2018; Hebert et al., 2018; Jacobsen et al., 2017; Quasdorf et al., 2017; Roberts et al., 2015; Thoft et al., 2021). Teamwork was a particularly important component of providing care, with staff acknowledging that “if colleagues get along with each other and collaborate well, this has a positive effect on the residents” (Thoft et al., 2021). In mixed-methods studies, when commenting on the success of an intervention, staff commented that “we did it together” (Hebert et al., 2018) which made the results even more satisfying. Some stated that it is not just important to get along with your colleagues, but “to listen to your colleagues’ experiences and learn from them” to support best practice (Jacobsen et al., 2017).

## Discussion

To the best of our knowledge, this systematic review and meta-analysis is the first to report available mixed-methods evidence on the delivery of person-centered care to people living with dementia in RAC. Our findings indicate that outcomes are highly variable. The evidence for person-centered care and its association with improved outcomes is limited. There were some signals in the descriptive quantitative data that person-centered care is positively associated with reduced agitation but the evidence for this was limited to five studies. All meta-analysis results were insignificant, but with narrow CIs and results trending toward significance. This creates challenges in making a recommendation regarding RAC practice and policy, yet provides a starting point to consider subjective resident concerns within person-centered care for people living with dementia.

Thirty-four frameworks, models of care, and programs were used to deliver person-centered care. Because of the number of different ways person-centered care was implemented (both within and across studies), it is challenging to interpret the consistency of results. For example, five studies provided person-centered care via Dementia Care Mapping, yet each of these studies yielded different results. Similarly, three of the studies that used the Montessori for Dementia and Ageing model of care were mixed methods; two focused on agitation (Roberts et al., 2015; van der Ploeg et al., 2013), and one focused on activities of daily living (Williams et al., 2013). The challenges of implementing person-centered care in RAC are recognized, with calls for a more robust instrument to measure the success of person-centered care interventions from the perspective of those affected (Fridberg et al., 2020). The new Implementation Framework for Aged Care was recently codesigned as a fit-for-purpose framework for embedding evidence into practice in aged care (Meyer et al., 2022). The Review of Innovative Models of Aged Care similarly acknowledges that the various methods of delivering person-centered care complicates evaluation (Dyer et al., 2019).

Agitation results varied greatly, reported to improve across four RCTs (Ballard et al., 2018; Chenoweth et al., 2009; Froggatt et al., 2020; Sloane et al., 2004), but not in two other RCTs (Rokstad et al., 2013; van der Ploeg et al., 2013). In fact, agitation results remained insignificant after meta-analysis. Other reviews have attempted to explain the prevalence and varied nature of agitation in people living with dementia, and why it can be complex to manage. One systematic review suggests that because nonpharmacologic interventions can take longer to be effective, they are difficult to implement in real-world settings (Ijaopo, 2017). A 2019 systematic review found that researchers are yet to quantify the impact of agitation on people living with dementia, and suggests that until this is clearly established, few interventions may be associated with a reduction in agitation (Anatchkova et al., 2019). Qualitative research has found that aged care staff find it challenging to manage agitation behaviors amongst residents with dementia due to impaired communication or comprehension of instructions (Watson & Hatcher, 2021), which may explain why staff did not perceive agitation levels of residents to change in the mixed-methods and qualitative studies (Booth et al., 2020; Kontos et al., 2016).

Neuropsychiatric symptoms were reported to improve across four RCTs (Ballard et al., 2016, 2018; Rokstad et al., 2013; Rosvik et al., 2014), but mixed-methods findings described residents spitting and hitting staff during the interventions (Kontos et al., 2016). The Neuropsychiatric Inventory Questionnaire, which was used to measure neuropsychiatric symptoms across the majority of studies in this review, has been criticized for being unable to accurately measure heterogeneity within neuropsychiatric symptoms, perhaps contributing to varied results (Lai, 2014).

Meta-analysis results for quality of life were also insignificant. A review of the availability and appropriateness of tools used in RAC settings found that the majority of quality of life measures do not have items relevant to RAC residents, such as control and autonomy. Instead, the quality-of-life measures used focused on physical strength and work, which may be less applicable (Courtney et al., 2003). A recently published systematic review further found that depression, functional impairment, and polypharmacy affect the quality of life of people living with dementia (Burks et al., 2021). These three elements are prevalent among older people living in RAC (Hillen et al., 2017), and may explain the lack of improvement in some studies. Mixed-methods research has been suggested as the best approach to assess the quality of life in RAC (Hall et al., 2011).

Qualitative findings of this review provided complementary data to the descriptive findings and meta-analysis. Time constraints were identified as a salient pattern to providing person-centered care; getting to know the resident and working within a collaborative staff environment were identified as enablers to providing person-centered care, all in line with existing literature (Kloos et al., 2020; Vassbo et al., 2019). These findings provide important insight, but people living with dementia should also be included in qualitative research (Webb et al., 2020). Qualitative research is important in this field, especially as the voice of the person with dementia is still absent from most quantitative tools (Edvardsson & Innes, 2010)

## Strengths and Limitations

This systematic review and meta-analysis incorporate quantitative and qualitative evidence focusing on person-centered care for people with dementia living in RAC. Further strengths include a comprehensive and systematic search of the literature, an examination of study design, quality of evidence, and outcome measures to compile the best-evidence base for this group.

We also acknowledge the review limitations. Meta-analysis results were remarkably heterogeneous and likely speak to the real-world challenges of implementing research in RAC, with different types of residents and staff members. The length of time that studies were conducted is a potential confounder, and we did not examine results relative to the length of time that studies were implemented. The relationship between resident outcomes and person-centered care may be influenced by factors that were not measured or reported, including the temporal relationship between dementia progression and comorbid conditions. Although this did not form part of our exclusion criteria, generalizability of results is potentially limited due to all studies being conducted in high-income countries. We also did not use search terms related to the subtypes of dementia, potentially limiting the number of retrieved articles. Results may not be transferable to specific RAC facilities, or low- and middle-income countries, where the prevalence of dementia is also high (Mattap et al., 2022). In addition to potential limitations associated with the implementation of this research, meta-analysis outcomes may be variable due to the clinometric differences between measurement tools employed across the various studies. Articles published in a language other than English were excluded from this review; as such, we may have missed results written in another language that address the research objectives.

## Future Directions

With an aging population and projected increase in the incidence and prevalence of dementia, we must build a strong evidence and understanding of how to best employ person-centered care to assist people living with dementia in RAC. This is especially important today, where health and social care systems globally are underfunded and understaffed. Directing limited resources to best-evidence care is likely to assist, as is including people living with dementia in shared decision making about their care.

## Conclusion

Although current evidence varies greatly in how person-centered care is implemented and affects people living with dementia in RAC, there are signals in the data to suggest that person-centered care is associated with some improved resident outcomes, particularly agitation and quality of life. Qualitative data provide individual staff perspectives and augment our understanding of what it means to deliver person-centered care in RAC. Additional research is required to inform tailored interventions that maximize the quality of life and improve resident outcomes more broadly.

## Supplementary Material

gnad052_suppl_Supplementary_Material

## Data Availability

The search strategy used is available to other researchers for replication purposes, and this data is made available in Supplementary Material. Studies reported in the manuscript had all undergone peer review and were published in academic journals. This study was preregistered with PROSPERO International Prospective Register of Systematic Reviews (registration number 106919).
