# Supplementary material for: The Delivery of Person-Centered Care for People Living With Dementia in Residential Aged Care: A Systematic Review and Meta-Analysis
Source: Gerontologist. 2023 May 5;64(5):gnad052. doi: 10.1093/geront/gnad052 (PMC11020247; doi:10.1093/geront/gnad052)
Supplement: gnad052_suppl_Supplementary_Material [file gnad052_suppl_supplementary_material.docx]

**Online Supplementary Material**

**Supplementary File 1: PRISMA Checklist**

Records identified from*:

Databases (n = 1109)

EMBASE (n = 622)

OVID MEDLINE (n = 229)

CINAHL (n = 192)

PSYCINFO (n = 67)

Records removed *before screening*:

Duplicate records removed (n = 77)

Records screened

(n = 1032)

Records excluded

(n = 898)

Reports sought for retrieval

(n = 133)

Reports not retrieved

(n = 0)

Reports assessed for eligibility

(n = 133)

Reports excluded: 82

Additional duplicates (n = 33)

Wrong study design (n = 26)

Not primary research (n = 19)

Not in English (n=4)

Studies included in review

(n = 51)

Reports of included studies

(n = 41)

**Identification**

**Screening**

**Included**

**Supplementary File 2: Medline Search Strategy**

**
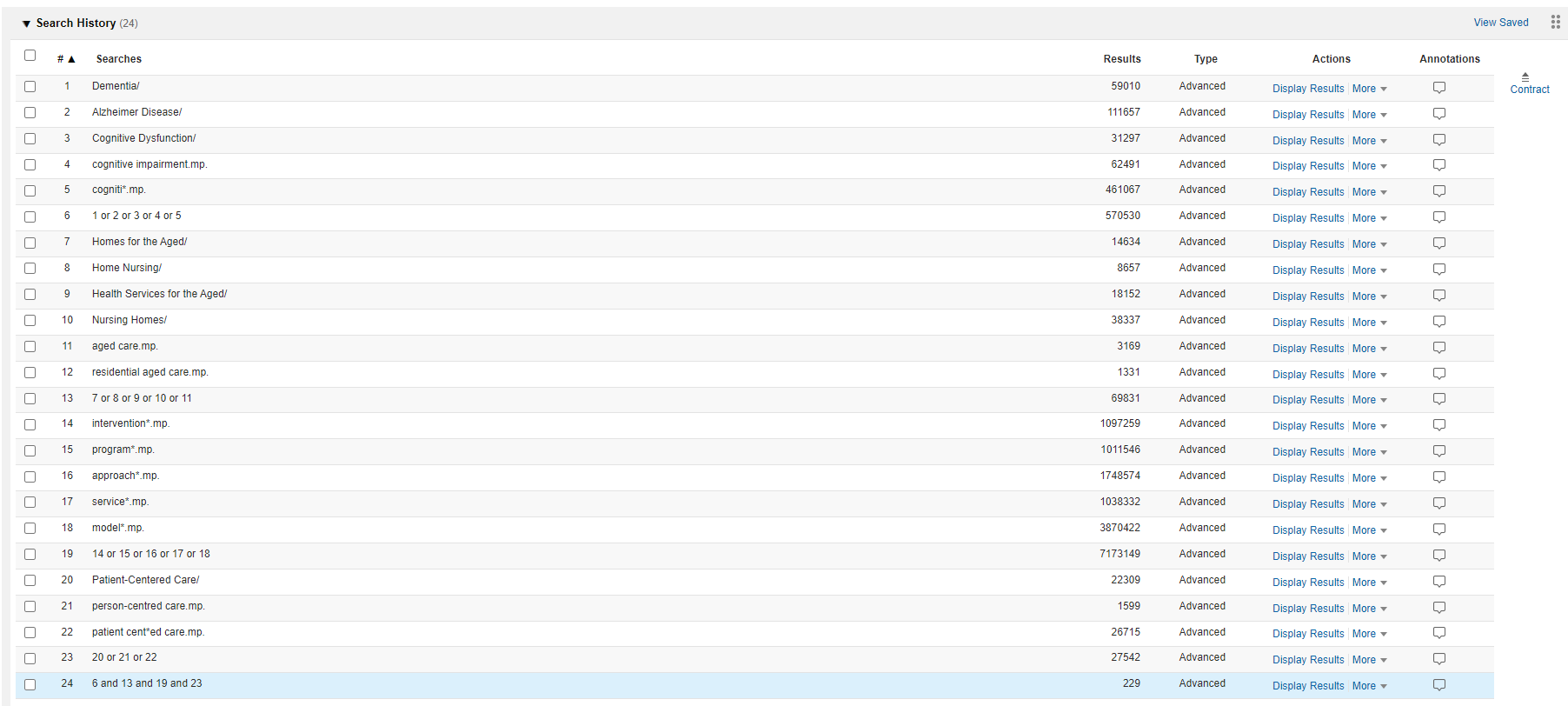
Medline**

**
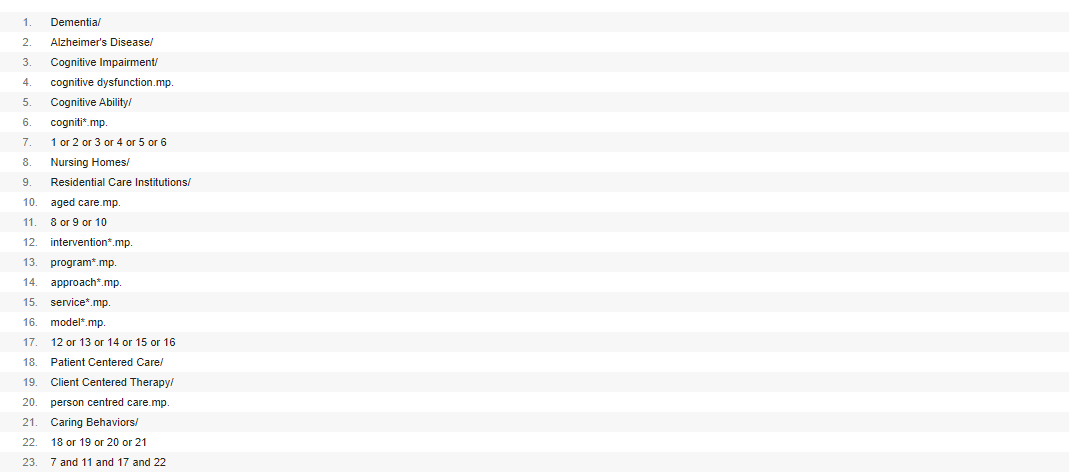
PsycInfo**

**EMBASE**


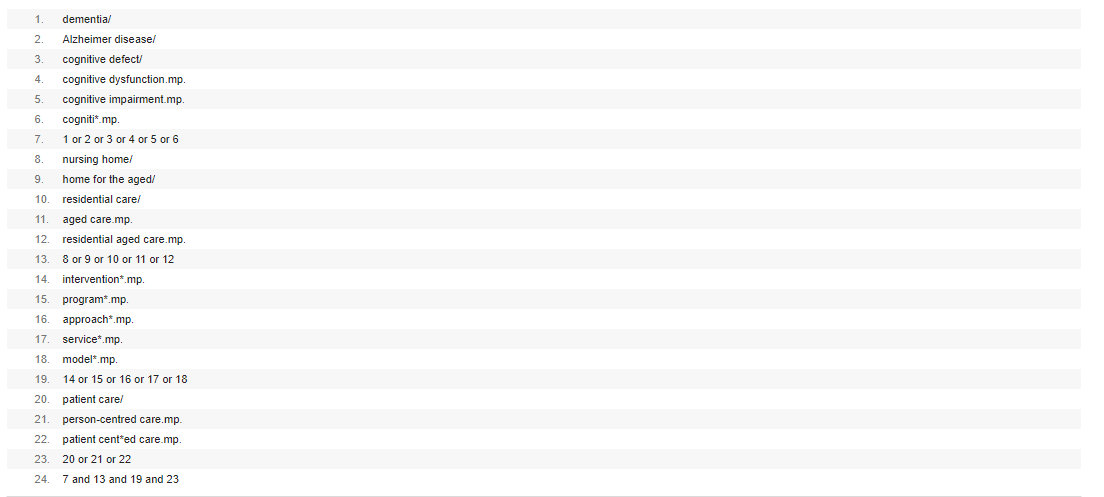


**CINAHL**


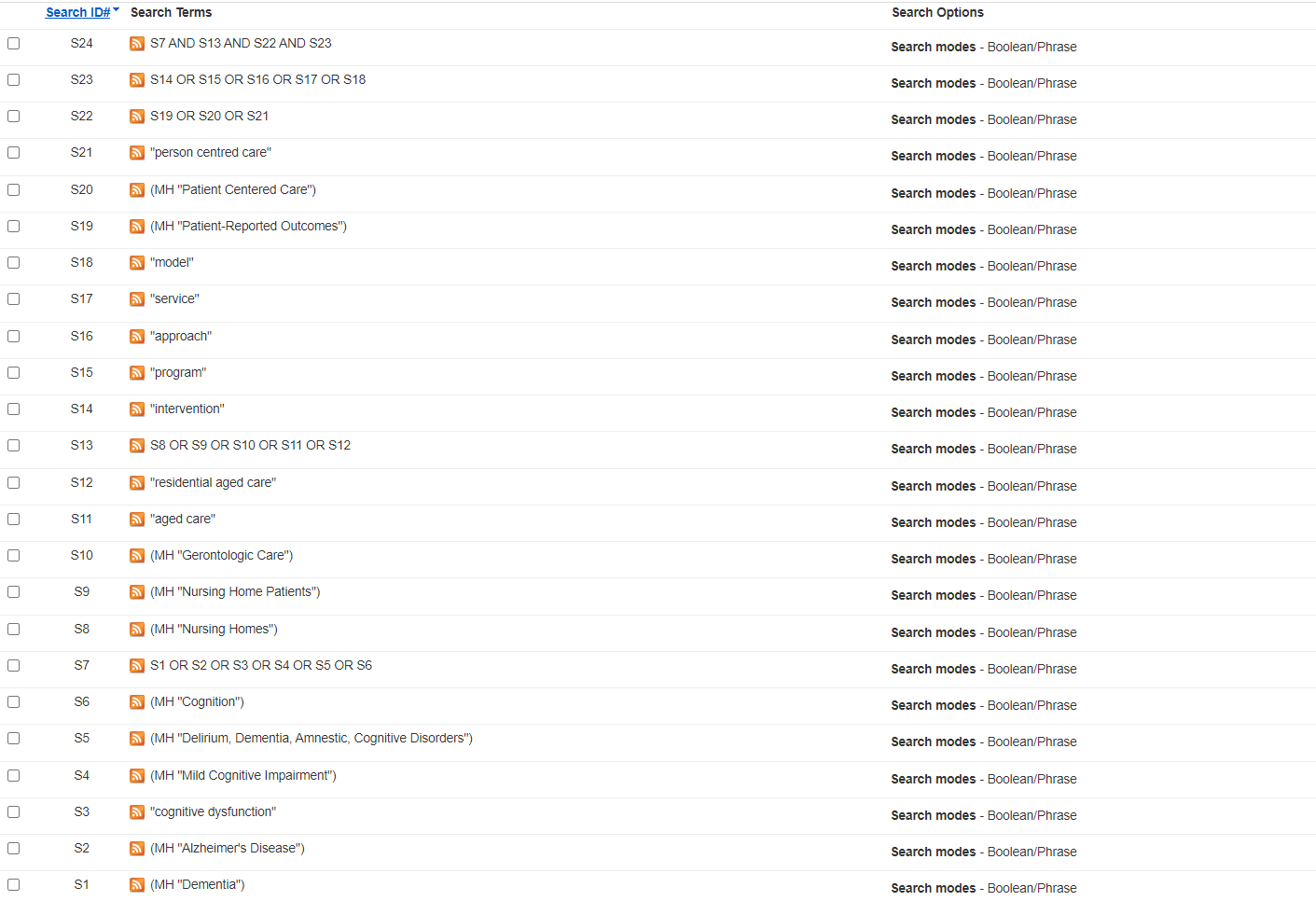


**Supplementary File 3: MMAT Results**

| **Study (Year)** | **Reviewer 1 Score** | **Reviewer 2 Score** | **Decision** |
| --- | --- | --- | --- |
| Boersma et al. (2017) | 5/5 | 5/5 | Include |
| Hebert et al. (2018) | 5/5 | 4/5 | Include |
| Jacobsen et al. (2017) | 4/5 | 4/5 | Include |
| Quasdorf et al. (2017) | 4/5 | 5/5 | Include |
| Roberts et al. (2015) | 2/5 | 2/5 | Include |
| Williams et al. (2015) | 4/5 | 4/5 | Include |

**Supplementary File 4: JBI Results**

| **Author** | **Year** | **Percentage** | **Reviewer 1 Rating** | **Include/Exclude** | **Percentage** | **Reviewer 2 Rating** | **Include/Exclude** |
| --- | --- | --- | --- | --- | --- | --- | --- |
| Ballard, Corbett, Orrell, Williams, Moniz-Cook, Romeo, Woods, Garrod, Testad, Woodward-Carlton, Wenborn, Knapp & Fossey | 2018 | 84.6 | High | Include | 84.6 | High | Include |
| Ballard, Orrell, Zhong, Moniz-Cook, Stafford, Whittaker, Woods, Corbett, Garrod, Khan, Woodward-Carlton, Wenborn & Fossey | 2016 | 84.6 | High | Include | 69.2 | Moderate | Include |
| Barbosa, Marques, Sousa, Nolan & Figueiredo | 2016 | 76.9 | Moderate | Include | 76.9 | Moderate | Include |
| Barbosa, Nolan, Sousa & Figueiredo (#47) | 2016 | 88.8 | High | Include | 80 | High | Include |
| Barbosa, Nolan, Sousa & Figueiredo (#50) | 2017 | 90% | High | Include | 76.9 | Moderate | Include |
| Barbosa, Nolan, Sousa & Figueiredo (#53) | 2016 | 76.9 | Moderate | Include | 76.9 | Moderate | Include |
| Berendonk, Kaspar, Bar & Hoben | 2019 | 69.2 | Moderate | Include | 69.2 | Moderate | Include |
| Bone, Cheung & Wade | 2010 | 33.3 | Low | Exclude | 33.3 | Low | Exclude |
| Booth, Zizzo, Robertson & Goodwin Smith | 2020 | 66.6 | Moderate | Include | 70 | Moderate | Include |
| Burack, Weiner & Reinhardt | 2012 | 66.6 | Moderate | Include | 90 | High | Include |
| Chapman & Toseland | 2007 | 46.2 | Low | Exclude | 46.2 | Low | Exclude |
| Chenoweth, Jeon, Stein-Parbury, Forbes, Fleming, Cook, Cheah, Fletcher & Tinslay | 2015 | 77 | Moderate | Include | 80 | High | Include |
| Chenoweth, King, Jeon, Brodaty, Stein-Parbury, Norman, Haas & Luscombe | 2009 | 80 | High | Include | 69.2 | Moderate | Include |
| Cherry, Carpenter, Waters, Hawkins, McGrew, Satterwhite, Stepien, Ruppelt, Herring | 2008 | NA | NA | Exclude - not primary data | NA | NA | Exclude - not primary data |
| Chu, Puts, Brooks, Parry & McGilton | 2020 | 55.5 | Moderate | Include | 88.9 | High | Include |
| Cooney, Hunter, Murphy, Casey, Devane, Smyth, Dempsey, Murphy, Jordan & O'Shea | 2014 | 30 | Low | Exclude | 80 | High | Include |
| Ducak, Denton & Elliott | 2018 | 80 | High | Include | 80 | High | Include |
| Froggatt, Bet, Bunn, Burnside, Coast, Dunleavy, Goodman, Hardwick, Jackson, Kinley, Lund, Lynch, Mitchell, Myring, Patel, Algorta, Preston, Scott, Silvera & Walshe | 2020 | 69.2 | Moderate | Include | 69.2 | Moderate | Include |
| Gillis, Lahaye, Dom, Lips, Arnouts & Van Bogaert | 2019 | 55.5 | Moderate | Include | 77.8 | Moderate | Include |
| Goodall, Tarldsen, Granbo & Serrano | 2021 | 100 | High | Include | 90 | High | Include |
| Goossens, Sevenants, Declercq & Van Audenhove | 2020 | 84.6 | High | Include | 53.8 | Moderate | Include |
| Halek, Dichter, Quasdorf, Riesner, Bartholemeyczik | 2013 | NA | NA | Excluded prior to appraisal - is a protocol paper | NA | NA | Excluded prior to appraisal - is a protocol paper |
| Halek, Reuther, Muller-Widmer, Trutschel & Holle | 2020 | 61.5 | Moderate | Include | 69.2 | Moderate | Include |
| Hoeffer, Talerico, Rasin, Mitchell, Stewart, McKenzie, Barrick, Rader & Sloane | 2006 | 53.8 | Moderate | Include | 53.8 | Moderate | Include |
| Joen, Luscombe, Chenoweth, Stein-Parbury, Brodaty & Haas | 2012 | 76.9 | Moderate | Include | 69.2 | Moderate | Include |
| Kontos, Mitcehll, Mistry & Ballon | 2010 | 80 | High | Include | 80 | High | Include |
| Kontos, Miller, Colobong, Palma, Binns, Low, Surr & Naglie | 2016 | 55.5 | Moderate | Include | 66.7 | Moderate | Exclude |
| Lawton, Van Haitsma, Klapper, Kelban, Katz & Corn | 1998 | 15.3 | Low | Exclude | 46.2 | Low | Exclude |
| Matthews, Farrell & Blackmore | 1996 | 55.5 | Moderate | Include | 66.7 | Moderate | Include |
| McNeil & Westphal | 2018 | 50 | Low | Exclude | 50 | Low | Exclude |
| Passalacqua & Harwood | 2012 | 55.5 | Moderate | Include | 66.7 | Moderate | Include |
| Resnick, Boltz, Galik, Fix, Holmes, Zhu & Barr | 2021 | 69.2 | Moderate | Include | 53.8 | Moderate | Include |
| Rokstad, Rosvik, Kirkevold, Selbaek, Benth & Engedal | 2013 | 76.9 | Moderate | Include | 61.5 | Moderate | Include |
| Rosvik, Engedal & Kirkevold | 2014 | 76.9 | Moderate | Include | 61.5 | Moderate | Include |
| Sloane, Hoeffer, Mitchell, McKenzie, Barrick, Rader, Stewart, Talerico, Rasin, Zink & Koch | 2004 | 53.8 | Moderate | Include | 53.8 | Moderate | Include |
| Stein-Parbury, Chenoweth, Jeon, Brodaty, Haas, Norman | 2012 | NA | NA | Excluded prior to appraisal - is a description of a model and has no primary data | NA | NA | Exclude |
| Swall, Hammar & Gransjon Craftman | 2020 | 80 | High | Include | 80 | High | Include |
| Thoft, Moller & Oller | 2021 | 60 | Moderate | Include | 60 | Moderate | Include |
| van der Ploeg, Eppingstall, Camp, Runci, Taffe & O'Connor | 2013 | 69.2 | Moderate | Include | 61.5 | Moderate | Include |
| Van Haitsma, Curyto, Abbott, Towsley, Spector & Kleban | 2015 | 84.6 | High | Include | 61.5 | Moderate | Include |
| Van Weert, Janssen, Van Dulment, Spreeuwenberg, Bensing & Ribbe | 2006 | 88.8 | High | Include | 88.9 | High | Include |
| Vezina, Robichaud, Voyer & Pelletier | 2011 | 40 | Low | Exclude | 30 | Low | Exclude |
| Williams, Perkhounkova, Jao, Bossen, Hein, Chung, Starykowicz & Turk | 2018 | 66.7 | Moderate | Include | 22.2 | Low | Exclude |
| Yasuda & Sakakibara | 2017 | 77.8 | Moderate | Include | 77.8 | Moderate | Include |
| Younger & Martin | 2000 | 22.2 | Low | Exclude | 22.2 | Low | Exclude |

| Supplementary File 5: Person-Centred Care Outcomes | | | | |
| --- | --- | --- | --- | --- |
| Author(s), Year | How long was the framework/model of care/program delivered for? | Who was data collected from? | Person-Centred Care Outcomes | Main findings |
| Ballard et al, 2016 | 9 months | - Residents | - Antipsychotic medication use - Understanding dementia - Understanding person-centred care - Neuropsychiatric symptoms | - Reduction in antipsychotic medication use (OR 0.17, 95% CI 0.05 – 0.60, p=0.006), although not statistically significant. - Reduced antipsychotic medication use by 50% - Significant effect on the interaction between antipsychotic review and social interaction (OR 2.06, 95% CI 1.06 – 4.01, p=0.04). - Significant effect on neuropsychiatric symptoms (p=0.05). |
| Ballard et al, 2018 | 9 months | - Residents - Staff | - Agitation - Quality of life - Neuropsychiatric symptoms | - Significant improvement in quality of life (Z Score 2.82, p=0.0042) - Significant improvement in agitation (Z Score 2.68, p=0.0076) - Significant improvement in neuropsychiatric symptoms (Z Score 3.52, p<0.001) |
| Barbosa et al, 2016 | 8 weeks | - Staff | - Understanding dementia - Understanding person-centred care - Shared decision-making and communication - Person-centred care environments - Stimulation - Staff training and caregiving | - Regarding participants’ verbal communicative behaviours, the frequency of the category “inform” increased significantly among dementia care workers in the experimental group (p<0.01). - The amount of laughs increased in dementia care workers in the experimental group (p<0.01). - There were positive, but not significant effects on the frequency and duration of “smile”, “resident-direct eye gaze”, and ‘affective touch”. |
| Barbosa et al, 2016 | 6 months | - Staff | - Understanding dementia - Understanding person-centred care - Shared decision-making and communication - Person-centred care environments - Stimulation - Staff training and caregiving | - At six months, dementia care workers were still more likely to “inform” and “laugh” with residents (p<0.01). - A significant time effect was found for the duration of “social conversation” (p<0.01). |
| Barbosa et al, 2017 | 8 weeks | - Staff | - Understanding dementia - Understanding person-centred care - Shared decision-making and communication - Person-centred care environments - Stimulation - Staff training and caregiving | - Results from the Global Behaviour Scale (a scale used to make global judgements about the quality of interactions) reported significant higher scores for staff undergoing the intervention. - However, a significant interaction effect was only found on one item – “put person before the task vs. put task before the person” – p=0.021. |
| Berendonk et al, 2019 | 6 weeks | - Staff - Residents | - Understanding dementia - Staff job satisfaction | - There was no difference in job dissatisfaction levels between staff participating in the intervention, and staff not participating in the intervention. - In the intervention group, the percentage of staff with greater job satisfaction increased during the study, although this was not statistically significant (p=0.053). - There were more reports submitted by staff related to residents’ emotional wellbeing participating in the intervention (p=0.027). |
| Boersma et al, 2017 | 21 months | - Staff | - Staff job satisfaction | - VCM contributed to staff job satisfaction, and applying VCM supported handling difficult resident behaviour and depressed mood of residents. - No significant differences in job satisfaction, time pressure, or social support from colleagues. |
| Burack et al, 2012 | 2 years | - Residents | - Agitation | - The intervention had a significant effect on forceful behaviours (p=0.022) and physical agitation (p=0.014) of residents. - There was no significant change in verbal agitation amongst residents (p=0.061). |
| Chenoweth et al, 2009 |  | - Residents | - Agitation - Antipsychotic medication use - Quality of life - Neuropsychiatric symptoms | - Agitation decreased within person-centred care during the study period (p=0.01). - The reduction in agitation for the dementia care mapping group was not significant (p=0.77). - Agitation of residents was significant lower in sites providing DCM (p=0.04) and person-centred care (p=0.01). - Participants in the person-centred care group were more likely to be prescribed anti-psychotic medication (0.34%) than in the DCM group (0.15%) or the usual care group (0.14%). - No statistically significant results for neuropsychiatric symptoms or quality of life. |
| Chu et al, 2020 | 6 months | - Residents | - Mobility - Activities of daily living - Quality of life | - Residents experienced a significant improvement in 2MWT from 53.60 to 81.07m, representing a 51.25% improvement. - Gait speed improvement by 55.11%. - Significant decline in quality of life during the control phase, which improved during the intervention phase (p=0.030). |
| Froggatt et al, 2020 | 24 weeks | - Staff - Residents | - Agitation - Quality of life - Neuropsychiatric symptoms | - Residents experienced improved agitation in the intervention group (mean 18.6, SD 5.5) compared to the control group (mean 25.3, SD 7.5), but statistical significance is not stated. - Residents experienced improved quality of life in the intervention group (mean 19.9, SD 7.5) compared to the control group (mean 28.1, SD 7.8), but statistical significance is not stated. - Residents experienced reduced neuropsychiatric symptoms in the intervention group (mean 1.1, SD 1.0) compared to the control group (mean 8.0, SD 4.7), but statistical significance is not stated. |
| Gillis et al, 2019 | 2 months | - Residents | - Agitation - Neuropsychiatric symptoms | - Significant effect on decreased aggression (p<0.001). - Significant effect on decreased depression (p=0.008). - Severity of neuropsychiatric symptoms decreased from 17.5 – 13.6, although not significant (p=.0.09). - As separate interventions, therapeutic touch (p=0.05), group music (p=0.04) and individual therapy (p<0.01) all decreased resident agitation. |
| Goossens, 2020 | 6 months | - Staff | - Shared decision-making and communication | - The level of supported decision-making increased significantly in the intervention group, and this effect was sustained at 6 months (p<0.0001). - At six months, staff perceived supported decision-making as an important part of their work (p=0.031), and felt more competent to make decisions (p=0.010). |
| Halek et al, 2020 | 7 months | - Residents - Staff | - Quality of life - Neuropsychiatric symptoms - Staff burnout | - Exploratory analysis revealed an 18% reduction in resident apathy, 29% reduction in eating disturbances, and 28% reduction in delusion. - Staff demonstrated a reduction in work-related burnout from the control to intervention phase, but this did not reach statistics significance. |
| Hoeffer et al, 2006 | 6 weeks | - Staff | - Understanding person-centred care | - Significant effect of intervention on gentleness during showering and towel bathing (p=0.01). - Significant effect of intervention on ease during showering and towel bathing (p=0.05). - No significant effect for staff on verbal support (p=0.064), confidence (p=0.197) or hassles (p=0.140). |
| Jacobsen et al, 2017 | 6 months | - Staff | - Understanding person-centred care - Shared decision-making and communication | - A slight increase in person-centred care measurements found that there was increased staff awareness related to not using restraints, although this did not reach statistics significant (p=0.078). |
| Jeon et al, 2012 | 2 years | - Staff | - Staff burnout - Neuropsychiatric symptoms (pertaining to staff) | - Significant effect of the intervention on emotional exhaustion of staff (p=0.006) - Significant effect on the general health of staff (p=0.033) |
| Kontos et al, 2016 | 12 weeks | - Residents - Staff | - Agitation - Quality of life - Neuropsychiatric symptoms - Staff training and caregiving | - Significant effect on neuropsychiatric symptoms (p=0.01). - Significant effect on quality of life (p<0.001). - Non-significant effect on agitation, although agitation did improve amongst residents (p=0.07). - Significant effect on occupational disruptiveness (p=0.02). |
| Matthews et al, 1996 | 12 weeks | - Residents | - Agitation | - Significant reduction in verbal agitation (p<0.01). - Daytime sleep increased significant amongst residents (p<0.01). - Night time sleep did not change significantly. |
| Passalacqua & Harwood, 2012 | 4 weeks | - Caregivers on behalf of residents | - Staff burnout (pertaining to caregivers) | - Residents spent significantly more time in leisure activities, easing the burden on caregivers (p=0.02). |
| Quasdorf | - |  |  |  |
| Resnick et al, 2021 | 12 months | - Residents | - Agitation - Shared decision-making and communication | - Significant decrease in agitation (p=0.045). - Significant decrease in depressive symptoms (p=0.003). - Significant decrease in resistance to care (p=0.04) - Significant difference in the quality of care interactions (p=0.001). |
| Roberts et al, 2015 | 18 months | - Staff - Residents - Family members | - Agitation - Antipsychotic medication use - Quality of life | - Significant reduction in agitation (p<0.0001). - Significant reduction in physically non-aggressive behaviour (p<0.0001). - Significant reduction in verbally agitated behaviour (p<0.0001). |
| Rokstad et al, 2013 | 1 year | - Residents | - Agitation - Neuropsychiatric symptoms - Quality of life | - No significant difference in agitation - Significant reduction in neuropsychiatric symptoms (p=0.04). - Significant improvement in quality of life (p=0.04). |
| Rosvik, 2014 | 10 months | - Residents | - Neuropsychiatric symptoms | - Significant reduction in neuropsychiatric symptoms (p=0.04). |
| Sloane et al, 2004 | 6 weeks | - Residents | - Agitation | - Significant reduction in agitation and aggression in the person-centred showering group (p<0.001) and in the towel bath group (p<0.001). - Less discomfort with towel bathing (p<0.003). - Average bath duration increased by a mean 3.3 minutes. |
| Van der Ploeg et al, 2013 | 4 weeks | - Residents | - Agitation | - In the Montessori group, agitation significantly improved during the intervention (mean 8.4, SD 9.9) compared to the control group (mean 10.0, SD 10.4). - After the intervention, there was no difference in agitation sustained between the two groups. |
| Van Haitsma et al, 2015 | 3 weeks | - Residents | - Activities of daily living | - Significant effect on ability to complete ADLs (p=0.0042). |
| Van Weert et al, 2006 | 18 months | - Staff | - Stimulation (multisensory, motor) - Staff training and caregiving | - Results showed a statistically significant increase in ‘Positive Person Work’. - Results showed a statistically significant decrease in ‘Malignant Social Psychology’. |
| Williams et al, 2015 | 9 months | - Residents | - Activities of daily living - Understanding person-centred care | - No significant impact on ability to perform activities of daily living. |
| Williams et al, 2018 | 3 months | - Staff - Residents | - Shared decision-making and communication | - Mean % time staff used elderspeak decreased from 28.5 – 19.6 (p=0.002). - Mean change in emotional not statistically significant (p=0.07). |
| Yasuda et al, 2017 | 2 months | - Residents | - Quality of life | - Significant improvement in quality of life (p<0.0001). |

ADL: activities of daily living; CI: confidence interval; DCM: dementia care mapping; OR: odds ratio; p: statistical significance (p<0.05 indicates significance); SD: standard deviation; VCM: vender contact method; Z-Score: statistical measure describing the value’s relationship to the mean; 2MWT: 2-minute walk test.

**Supplementary File 6: Qualitative Quotes**

| ***Barriers to Providing Person-Centred Care*** | | |
| --- | --- | --- |
| *Authors* | *Theme* | *Representative Quote* |
| Booth | Behaviours | They’re showing behaviours because their needs are not met, they are frustrated, they feel bored, and they have no control |
| Kontos | Behaviours | We have a lot of residents on our floor…they’re gonna lash out they’re gonna probably spit on you, try to hit you, they’ll get aggressive |
|  | Time | But sometimes, especially in the morning you have to rush rush rush. Sometimes I said ‘Oh my god, I remember my education’, the one [from the video] that don’t…rush residents because if you rush them it will affect you, |
| Boersma | Time | Indeed, and that … this lady has a lot of pain and you have to deal with this as best you can. So it really becomes a matter of, you know, slow down… Spend all your time on her, and not think about ‘but I still have five more residents to go’. You know, you have to let that go |
| Ducak | Time | stems from staff needs, hours of service, how they document what they do with their time, how fast things have to get done, their schedule, keeping to the Ministry of Health standards, you know, so many people have to be toileted and bathed and everything else |
|  | Task-oriented | Some of the barriers [are], you know, nursing being task-oriented and thinking that the Ministry is not going to allow for this |
|  | Task-oriented | Staff buy-in is another problem where I go in and I say, ‘‘I would like you to try this.’’ And they’re like, ‘‘Well, I have to get eleven people out of bed. I’m not doing that for him.’’ It’s a very task-oriented work environment and so to add something that is sort of unique or a little bit out of the box is threatening to some of the staff |
|  | RAC medicalised | We’re just dealing with a primarily medical environment, right? And, while even the government’s trying to get away from that it’s hard when you’re dealing with people who have been in nursing for 20 years and suddenly be open to this whole other way of nursing. |
|  | Family resistance | we did receive a lot of resistance from certain families because they expect to come in, and the program staff have someone playing a piano and everyone’s singing and dancing and piled in a big room all the time. And it’s recognizing that do you do that in your own life? Like, do you do that every day?...Because then with our [staffing] ratios we’re not providing that quality programming to everybody else that doesn’t attend. |
|  | Staff shortages | our dementia unit is quite active right at the moment and at this point it’s attention span and staffing levels. Because there’s only one activation person to 28 [residents] |
|  | English as a second language | for some English is a second language |
| Thoft | Family resistance | ‘So, we are in such a predicament right now where it is difficult for the relatives to get it done. (…) some are obviously challenged because they are tired and worn out’. |
| Goodall | Psychosocial health ignored | I think the majority of therapy interventions or healthcare is oriented to physical aspects, and the psychological aspects are not taken very well care of… this causes a lot of problems. |
| ***Enablers to Providing Person-Centred Care*** | | |
| Booth | Helping residents adjust to living in RAC | Person 2 didn’t want to be involved at first when she arrived. She was trying to open doors and get out, trying to get to work or staying in her room. She will now join in and come after dinner for activities.’ |
|  | Getting to know the resident | Take the extra 5 minutes to encourage her and she will respond |
|  | Encouraging relationships between residents | He is interested in what’s happening and tells others they’re doing well. He smiles and then claps, and behaves like a cheerleader ... Talks encouragingly to the other residents in response to their efforts, praises others, cheers them on |
| Chenoweth | Staff collaboration | The care staff learned to relax, break down the task (at times of resident resistance to care) . . . like changing the pad first and showering later . . . supervising instead of intervening and controlling . . . this works better for them both and both are happy. |
| Ducak | Encouraging relationships between residents | They initiate conversation on their own more. And they have a higher level of engagement during the activity as well. ... they’re much more engaged, content, not so apathetic or isolating themselves |
|  | Staff collaboration | it’s getting everybody’s buy-in that they’re all responsible |
| Goodall | Getting to know the resident | The more we learn about our residents, the more we can provide care |
| Herbert | Staff collaboration | “We did it together, didn’t we?” |
| Jacobsen | Staff collaboration | “Since we were all there sitting together we discussed and listened to each other. It is very important to listen to your colleagues’ experiences and learn from them |
| Kontos | Getting to know the resident | Knowing the interests of the residents prior to them having dementia, it does help |
| Quasdorf | Staff collaboration | We do have staff members who like to be here. That’s the way it is |
| Roberts | Staff collaboration | the sense of homeliness and trust that exists in (the Unit) surely marks leaders and staff as outstanding in their duties |
|  | Getting to know the resident | Making patients feel wanted, it feels like home |
| Swall | Getting to know the resident | We had a lady in our ward, and she was …, she never wanted to get out of bed … we knew that she liked music, so I came in and sang one time, and she started singing with me. She sang along very well |
| Thoft | Staff collaboration | We now work together as a team much better, and yes, I think if colleagues get along with each other, collaborate well, this has a positive effect on the residents, and then you enjoy your work more |
|  | Getting to know the resident | ‘It’s important that you know the residents' life stories… otherwise you don’t know how to help them |
| Williams | Helping residents adjust to living in RAC | Person 2 didn’t want to be involved at first when she arrived. She was trying to open doors and get out, trying to get to work or staying in her room. She will now join in and come after dinner for activities.’ |

RAC: residential aged care

**Supplementary File 7. PRISMA 2020 Checklist**

| **Section and Topic** | **Item #** | **Checklist item** | **Location where item is reported (Page)** |
| --- | --- | --- | --- |
| **TITLE** | | |  |
| Title | 1 | Identify the report as a systematic review. | 1 |
| **ABSTRACT** | | |  |
| Abstract | 2 | See the PRISMA 2020 for Abstracts checklist. | 2 |
| **INTRODUCTION** | | |  |
| Rationale | 3 | Describe the rationale for the review in the context of existing knowledge. | 3-4 |
| Objectives | 4 | Provide an explicit statement of the objective(s) or question(s) the review addresses. | 4 |
| **METHODS** | | |  |
| Eligibility criteria | 5 | Specify the inclusion and exclusion criteria for the review and how studies were grouped for the syntheses. | 5 |
| Information sources | 6 | Specify all databases, registers, websites, organisations, reference lists and other sources searched or consulted to identify studies. Specify the date when each source was last searched or consulted. | 5-6 |
| Search strategy | 7 | Present the full search strategies for all databases, registers and websites, including any filters and limits used. | 5, Supplementary Material |
| Selection process | 8 | Specify the methods used to decide whether a study met the inclusion criteria of the review, including how many reviewers screened each record and each report retrieved, whether they worked independently, and if applicable, details of automation tools used in the process. | 5-6 |
| Data collection process | 9 | Specify the methods used to collect data from reports, including how many reviewers collected data from each report, whether they worked independently, any processes for obtaining or confirming data from study investigators, and if applicable, details of automation tools used in the process. | 5-7 |
| Data items | 10a | List and define all outcomes for which data were sought. Specify whether all results that were compatible with each outcome domain in each study were sought (e.g. for all measures, time points, analyses), and if not, the methods used to decide which results to collect. | 6-7 |
|  | 10b | List and define all other variables for which data were sought (e.g. participant and intervention characteristics, funding sources). Describe any assumptions made about any missing or unclear information. | 6-7 |
| Study risk of bias assessment | 11 | Specify the methods used to assess risk of bias in the included studies, including details of the tool(s) used, how many reviewers assessed each study and whether they worked independently, and if applicable, details of automation tools used in the process. | 6-7 |
| Effect measures | 12 | Specify for each outcome the effect measure(s) (e.g. risk ratio, mean difference) used in the synthesis or presentation of results. | 7 |
| Synthesis methods | 13a | Describe the processes used to decide which studies were eligible for each synthesis (e.g. tabulating the study intervention characteristics and comparing against the planned groups for each synthesis (item #5)). | 7 |
|  | 13b | Describe any methods required to prepare the data for presentation or synthesis, such as handling of missing summary statistics, or data conversions. | 7 |
|  | 13c | Describe any methods used to tabulate or visually display results of individual studies and syntheses. | 7 |
|  | 13d | Describe any methods used to synthesize results and provide a rationale for the choice(s). If meta-analysis was performed, describe the model(s), method(s) to identify the presence and extent of statistical heterogeneity, and software package(s) used. | 7 |
|  | 13e | Describe any methods used to explore possible causes of heterogeneity among study results (e.g. subgroup analysis, meta-regression). | 7 |
|  | 13f | Describe any sensitivity analyses conducted to assess robustness of the synthesized results. | 7 |
| Reporting bias assessment | 14 | Describe any methods used to assess risk of bias due to missing results in a synthesis (arising from reporting biases). | 7 |
| Certainty assessment | 15 | Describe any methods used to assess certainty (or confidence) in the body of evidence for an outcome. | 7 |
| **RESULTS** | | |  |
| Study selection | 16a | Describe the results of the search and selection process, from the number of records identified in the search to the number of studies included in the review, ideally using a flow diagram. | 8, Supplementary File 1 |
|  | 16b | Cite studies that might appear to meet the inclusion criteria, but which were excluded, and explain why they were excluded. | 8, Supplementary File 1 |
| Study characteristics | 17 | Cite each included study and present its characteristics. | 8-9, Table 1 |
| Risk of bias in studies | 18 | Present assessments of risk of bias for each included study. | 10, Supplementary File 3, 4 |
| Results of individual studies | 19 | For all outcomes, present, for each study: (a) summary statistics for each group (where appropriate) and (b) an effect estimate and its precision (e.g. confidence/credible interval), ideally using structured tables or plots. | 14, Figure 1, 2, 3 |
| Results of syntheses | 20a | For each synthesis, briefly summarise the characteristics and risk of bias among contributing studies. | 10, 14, Supplementary File 3, 4 |
|  | 20b | Present results of all statistical syntheses conducted. If meta-analysis was done, present for each the summary estimate and its precision (e.g. confidence/credible interval) and measures of statistical heterogeneity. If comparing groups, describe the direction of the effect. | 14, Figure 1, 2, 3 |
|  | 20c | Present results of all investigations of possible causes of heterogeneity among study results. | 14 |
|  | 20d | Present results of all sensitivity analyses conducted to assess the robustness of the synthesized results. | N/A |
| Reporting biases | 21 | Present assessments of risk of bias due to missing results (arising from reporting biases) for each synthesis assessed. | Supplementary File 3, 4 |
| Certainty of evidence | 22 | Present assessments of certainty (or confidence) in the body of evidence for each outcome assessed. | 14 |
| **DISCUSSION** | | |  |
| Discussion | 23a | Provide a general interpretation of the results in the context of other evidence. | 17 |
|  | 23b | Discuss any limitations of the evidence included in the review. | 18-19 |
|  | 23c | Discuss any limitations of the review processes used. | 18-19 |
|  | 23d | Discuss implications of the results for practice, policy, and future research. | 17, 19 |
| **OTHER INFORMATION** | | |  |
| Registration and protocol | 24a | Provide registration information for the review, including register name and registration number, or state that the review was not registered. | 5 |
|  | 24b | Indicate where the review protocol can be accessed, or state that a protocol was not prepared. | 5 |
|  | 24c | Describe and explain any amendments to information provided at registration or in the protocol. | N/A |
| Support | 25 | Describe sources of financial or non-financial support for the review, and the role of the funders or sponsors in the review. | 1 |
| Competing interests | 26 | Declare any competing interests of review authors. | 1 |
| Availability of data, code and other materials | 27 | Report which of the following are publicly available and where they can be found: template data collection forms; data extracted from included studies; data used for all analyses; analytic code; any other materials used in the review. | 5, Supplementary File 2, 3, 4 |

*From:*  Page MJ, McKenzie JE, Bossuyt PM, Boutron I, Hoffmann TC, Mulrow CD, et al. The PRISMA 2020 statement: an updated guideline for reporting systematic reviews. BMJ 2021;372:n71. doi: 10.1136/bmj.n71

For more information, visit: <http://www.prisma-statement.org/>
